# Role of the Nucleus Accumbens in Signaled Avoidance Actions

**DOI:** 10.1523/ENEURO.0314-24.2024

**Published:** 2024-10-10

**Authors:** Ji Zhou, Sebastian Hormigo, Muhammad S. Sajid, Manuel A. Castro-Alamancos

**Affiliations:** Department of Neuroscience, University of Connecticut School of Medicine, Farmington, Connecticut 06001

**Keywords:** avoidance, midbrain, movement, nucleus accumbens, sensorimotor

## Abstract

Animals, humans included, navigate their environments guided by sensory cues, responding adaptively to potential dangers and rewards. Avoidance behaviors serve as adaptive strategies in the face of signaled threats, but the neural mechanisms orchestrating these behaviors remain elusive. Current circuit models of avoidance behaviors indicate that the nucleus accumbens (NAc) in the ventral striatum plays a key role in signaled avoidance behaviors, but the nature of this engagement is unclear. Evolving perspectives propose the NAc as a pivotal hub for action selection, integrating cognitive and affective information to heighten the efficiency of both appetitive and aversive motivated behaviors. To unravel the engagement of the NAc during active and passive avoidance, we used calcium imaging fiber photometry to examine NAc GABAergic neuron activity in *ad libitum* moving mice performing avoidance behaviors. We then probed the functional significance of NAc neurons using optogenetics and genetically targeted or electrolytic lesions. We found that NAc neurons code contraversive orienting movements and avoidance actions. However, direct optogenetic inhibition or lesions of NAc neurons did not impair active or passive avoidance behaviors, challenging the notion of their purported pivotal role in adaptive avoidance. The findings emphasize that while the NAc encodes avoidance movements, it is not required for avoidance behaviors, highlighting the distinction between behavior encoding or representation and mediation or generation.

## Significance Statement

Adaptive avoidance behaviors are used by animals, including humans, to avert danger in the environment. For example, humans use signaled avoidance strategies to cross the street at a crosswalk. This study sheds light on the role of an area in the forebrain called the nucleus accumbens (NAc) purported to be important for adaptive avoidance behaviors. The findings show that like many other areas, NAc is engaged during these behaviors but is not required to generate them. The results open new avenues for understanding the neural basis of adaptive avoidance behaviors and the functional role of NAc.

## Introduction

Animals are often guided by sensory cues to engage in purposeful movements motivated by various contingencies. The execution of avoidance behaviors in response to specific signals represents an adaptive strategy employed in situations where impending danger is indicated. For example, pedestrians navigate a crosswalk when prompted by signals, thereby avoiding potential harm from oncoming traffic. Despite the inherent risk associated with such avoidance behavior, individuals typically execute it routinely, characterized by a sense of caution rather than fear, owing to the perceived control and predictability of the situation (Kamin et al., 1963; Mineka, 1979; Zhou et al., 2022). The exploration of adaptive active avoidance behavior has a rich history spanning nearly a century (Mowrer and Lamoreaux, 1942). However, despite decades of research, a consensus on the neural mechanisms underlying adaptive avoidance remains elusive.

Models outlining the circuitry of active avoidance draw inspiration from the pavlovian fear conditioning circuit (Davis, 1997; LeDoux, 2000; Maren, 2001; Fanselow and Gale, 2003; Kim and Jung, 2006). In this framework, the conditioned stimulus (CS) traverses from the thalamus to the lateral nucleus of the amygdala, where it forms an association with the aversive stimulus. Subsequently, the signal progresses to the central nucleus, inducing conditioned responses like freezing and potentiated startle. During active avoidance scenarios, a modification of this circuitry is proposed. The CS travels from the lateral nucleus of the amygdala to the basal nucleus and onward to the nucleus accumbens (NAc) under the regulatory influence of the prefrontal cortex (Amorapanth et al., 2000; Moscarello and LeDoux, 2013; Bravo-Rivera et al., 2014; Ramirez et al., 2015). Other theoretical frameworks consider various interacting systems—activation, inhibition, and fight–flight–freeze—as coordinators of avoidance responses (Konorski, 1967; Dickinson et al., 1980; Gray and McNaughton, 2000; Dickinson and Balleine, 2002; McNaughton and Corr, 2004). In this perspective, the NAc is identified as a component of the activating system that drives active responses. In either framework, the NAc assumes a pivotal role in the orchestration of active avoidance behaviors.

The NAc integrates excitatory signals from key forebrain regions, including the medial prefrontal cortex, basolateral amygdala, hippocampus, and thalamus. This intricate interplay is modulated by dopaminergic and GABAergic inputs from the ventral tegmental area (Gerfen and Surmeier, 2011). Predominantly, NAc is composed of GABAergic medium spiny output neurons, while a small fraction (∼5%) of GABAergic interneurons, some releasing acetylcholine, contribute to the neural dynamics within the NAc (Castro and Bruchas, 2019). Various neurochemical and receptor markers delineate the NAc into core and shell regions, each comprising patch and matrix areas housing both direct and indirect pathway medium spiny neurons (Gerfen and Surmeier, 2011; Castro and Bruchas, 2019). The direct pathway influences the substantia nigra and ventral tegmental area in the midbrain, whereas the indirect pathway targets the pallidum and hypothalamus, which subsequently project to the midbrain. These cell groups have been intensely studied revealing distinct features but activate concurrently during behavior (Cui et al., 2013; Klaus et al., 2019). Intriguingly, part of the direct pathway reaches an area in the midbrain pedunculopontine tegmentum (PPT) that is critical for active avoidance, and excitation of these direct pathway GABAergic fibers inhibits PPT and blunts active avoidance (Hormigo et al., 2019), supporting the notion that NAc may play an important role in active avoidance.

Traditionally, the function of the NAc has been closely associated with its dopamine innervation, acting as a mediator of the hedonic aspects of the reward system that propels approach responses. However, this conceptualization has faced challenges, giving way to an alternative framework where the NAc assumes a central role in action selection, integrating cognitive and affective information from various afferent regions. In doing so, it enhances the efficiency and vigor of both appetitive and aversive motivated behaviors (Berridge, 2007; Nicola, 2007; Yin et al., 2008; Salamone and Correa, 2012; Saunders and Robinson, 2012; Floresco, 2015). From the perspective of appetitive, approach, or reward-seeking behavior, the NAc is recognized as a crucial component of an activation and effort-related motivational circuit, influenced by dopamine (Mogenson et al., 1980; Salamone and Correa, 2023). However, a notable gap remains in understanding its purported role during avoidance behaviors, where active or passive responses are motivated by the anticipation of harmful and punishing consequences.

To test the engagement of the NAc during active and passive avoidance behaviors, we measured NAc neuron activity in *ad libitum* moving mice using calcium imaging fiber photometry. Additionally, we utilized optogenetics and electrolytic and genetically targeted lesions to probe the functional significance of these neurons during avoidance behaviors. Our findings reveal that NAc neurons as a whole code various aspects of signaled avoidance behaviors but are not required to generate these behaviors.

## Materials and Methods

### Experimental design and statistical analysis

The methods used in the present paper are like those employed in a previous study (Hormigo et al., 2023). Consequently, the description of the results follows a similar text format. All procedures were reviewed and approved by the Institutional Animal Care and Use Committee and conducted in adult (>8 weeks) male and female mice. Experiments involved a repeated-measure (RM) design in which the mice or cells serve as their own controls (comparisons within groups), but we also compared experimental groups of mice or cells between groups (comparisons between groups). For comparisons within groups, we tested for a main effect of a variable (e.g., stimulus) using a RMANOVA or a linear mixed-effect model with fixed effects and random effects (e.g., sessions nested within the subjects as per data ∼ stimulus + (1|subjects/sessions lme4 syntax in R) followed by comparisons with Tukey's test. For comparisons between different groups, we used the same approach but included the group as an additional fixed effect [data ∼ group * stimulus + (1|subjects/sessions)]. Using the standard errors derived from the model, Tukey’s tests were conducted for the effect of the fixed-effect factors (within-group comparisons) or for the group–stimulus interaction (between group comparisons). We report the Tukey’s values for the relevant multiple comparisons. Plots report mean ± SEM unless otherwise indicated.

To enable rigorous approaches, we maintain a centralized metadata system that logs all details about the experiments and is engaged for data analyses (Castro-Alamancos, 2022). Moreover, during daily behavioral sessions, computers run experiments automatically using preset parameters logged for reference during analysis. Analyses are performed using scripts that automate all aspects of data analysis from access to logged metadata and data files to population statistics and graph generation.

### Strains and adeno-associated viruses (AAVs)

To record from NAc GABAergic neurons using calcium imaging, we injected a Cre-dependent AAV [AAV5-syn-FLEX-jGCaMP7f-WPRE (Addgene, 7 × 10^12^ vg/ml)] in the NAc of Vgat-cre mice [Jax 028862; B6J.129S6(FVB)-Slc32a1^tm2(cre)Lowl^/MwarJ] to express GCaMP6f/7f. An optical fiber was then placed in this location. To inhibit NAc GABAergic neurons using optogenetics, we expressed eArch3.0 by injecting AAV5-EF1a-DIO-eArch3.0-EYFP (UNC Vector Core; titers, 3.4 × 10^12^ vg/ml) in the NAc of Vgat-cre mice. In addition, to inhibit NAc neurons, we expressed ChR2 in cholinergic neurons of the NAc, which are GABAergic and inhibit principal GABAergic neurons locally, by injecting AAV5-EF1a-DIO-hChR2(H134R)-eYFP (UPenn Vector Core or Addgene; titers, 1.8 × 10^13^ GC/ml) into the NAc of Chat-cre mice (Jax 031661; B6.129S-Chat^tm1(cre)Lowl^/MwarJ). No-opsin controls were injected with AAV8-hSyn-EGFP (Addgene; titers, 4.3 × 10^12^ GC/ml by quantitative PCR) or nil in the NAc.

As additional controls of the optogenetic effects of manipulating the activity of GABAergic (Vgat) NAc neurons, we targeted Vglut2 or CaMKII in NAc. For Arch controls, we injected AAV5-EF1a-DIO-eArch3.0-EYFP (UNC Vector Core; titers, 3.4 × 10^12^ vg/ml) in the NAc of Vglut2-cre mice and injected AAV5-CaMKIIa-eArchT3.0-EYFP (UNC Vector Core; titers, 4 × 10^12^ vg/ml) in the NAc of C57BL/6J mice. In accordance with the lack of glutamatergic (e.g., Vglut2) cells in NAc, these controls resulted in nil expression in the NAc (Vong et al., 2011).

For optogenetics, we implanted dual optical fibers bilaterally in the NAc. All the AAVs and optogenetic methods used in the present study have been validated in previous studies using slice and/or in vivo electrophysiology (Hormigo et al., 2016, 2019, 2021a,c).

### Surgeries

Optogenetics and fiber photometry experiments involved injecting 0.2–0.4 µl AAVs per site during isoflurane anesthesia (∼1%). Animals received carprofen after surgery. The stereotaxic coordinates for injection in NAc are (in mm from the bregma; lateral from the midline; ventral from the bregma–lambda plane) 1.2 anterior, 0.8, and 4.3. In these experiments, a single (400 µm in diameter for fiber photometry) or dual (200 µm in diameter for optogenetics) optical fiber was implanted unilaterally or bilaterally during isoflurane anesthesia. The stereotaxic coordinates for the implanted optical fibers (in mm) in the NAc are 1.2 anterior, 1, and 4.3. No-opsin mice were implanted with cannulas in NAc or adjacent sites.

Bilateral electrolytic lesions were performed by lowering an electrode (0.25 mm diameter, insulated stainless steel with a 0.5 mm exposed tip) into the NAc and applying current (0.6 mA) for 15 s in an anterior (in mm: 1.0 anterior, 0.8 lateral, and 4.6 ventral) and a posterior site (1.6 anterior, 0.8, and 4.6 mm) per side.

### Active avoidance tasks

Mice were trained in a signaled active avoidance task, as previously described (Hormigo et al., 2016, 2019). During an active avoidance session, mice are placed in a standard shuttle box (16.1″ × 6.5″) that has two compartments separated by a partition with side walls forming a doorway that the animal must traverse to shuttle between compartments. A speaker is placed on one side, but the sound fills the whole box, and there is no difference in behavioral performance (signal detection and response) between sides. A trial consists of a 7 s avoidance interval followed by a 10 s escape interval. During the avoidance interval, an auditory CS (8 kHz, 85 dB) is presented for the duration of the interval or until the animal produces a conditioned response (avoidance response) by moving to the adjacent compartment, whichever occurs first. If the animal avoids, by moving to the next compartment, the CS ends, the escape interval is not presented, and the trial terminates. However, if the animal does not avoid, the escape interval ensues by presenting white noise and a mild scrambled electric footshock (0.3 mA) delivered through the grid floor of the occupied half of the shuttle box. This unconditioned stimulus (US) readily drives the animal to move to the adjacent compartment (escape response), at which point the US terminates, and the escape interval and the trial ends. Thus, an “avoidance response” will eliminate the imminent presentation of a harmful stimulus. An “escape response” is driven by presentation of the harmful stimulus to eliminate the harm it causes. Successful avoidance warrants the absence of harm. Each trial is followed by an intertrial interval (duration is randomly distributed; 25–45 s range), during which the animal awaits the next trial. We employed four variations of the basic signaled active avoidance procedure termed AA1, AA2, AA3, and AA4.

In AA1, mice are free to cross between compartments during the intertrial interval; there is no consequence for intertrial crossings (ITCs).

In AA2, mice receive a 0.2 s footshock (0.3 mA) and white noise for each ITC. Therefore, in AA2, mice must passively avoid during the intertrial interval by inhibiting their tendency to shuttle between trials. Thus, during AA2, mice perform both signaled active avoidance during the signaled avoidance interval (like in AA1) and unsignaled passive avoidance during the unsignaled intertrial interval.

In AA3, mice are subjected to a CS discrimination procedure in which they must respond differently to a CS1 (8 kHz tone at 85 dB) and a CS2 (4 kHz tone at 70 dB) presented randomly (half of the trials are CS1). Mice perform the basic signaled active avoidance to CS1 (like in AA1 and AA2) but also perform signaled passive avoidance to CS2, and ITCs are not punished. In AA3, if mice shuttle during the CS2 avoidance interval (7 s), they receive a 0.5 s footshock (0.3 mA) with white noise and the trial ends. If animals do not shuttle during the CS2 avoidance interval, the CS2 trial terminates at the end of the avoidance interval (i.e., successful signaled passive avoidance).

In AA4, three different CS's, CS1 (8 kHz tone at 81 dB), CS2 (10 kHz tone at 82 dB), and CS3 (12 kHz tone at 82 dB), signal a different avoidance interval duration of 4, 7, and 15 s, respectively. Like in AA2, mice are punished for producing ITCs. In AA4, mice adjust their response latencies according to the duration of the avoidance interval signaled by each CS.

There are three main variables representing task performance. The percentage of active avoidance responses (% avoids) represents the trials in which the animal actively avoided the US in response to the CS. The response latency (latency) represents the time (seconds) at which the animal enters the safe compartment after the CS onset; avoidance latency is the response latency only for successful active avoidance trials (excluding escape trials). The number of crossings during the intertrial interval (ITCs) represents random shuttling due to locomotor activity in the AA1 and AA3 procedures or failures to passively avoid in the AA2 procedure. The sound pressure level (SPL) of the auditory CS's was measured using a microphone (PCB Piezotronics 377C01) and amplifier (X100) connected to a custom LabVIEW application that samples the stimulus within the shuttle cage as the microphone rotates driven by an actuator controlled by the application.

### Fiber photometry

We employed a two-channel excitation (465 and 405 nm) and two-channel emission (525 and 430 nm for GCaMP6f and control emissions) fiber photometry system (Doric Lenses). Alternating light pulses were delivered at 100 Hz (per each 10 ms, 465 is on for 3 ms, and 2 ms later 405 is on for 3 ms). While monitoring the 525 nm emission channel, we set the 465 light pulses in the 20–60 µW power range, and then the power of the 405 light pulses was adjusted (20–50 µW) to approximately match the response evoked by the 465 pulses. During recordings, the emission peak signals evoked by the 465 (GCaMP6f) and 405 (isobestic) light pulses were acquired at 5–20 kHz and measured at the end of each pulse. To calculate *F*_o_, the measured peak emissions evoked by the 405 nm pulses were scaled to those evoked by the 465 pulses (*F*) using the slope of the linear fit. Finally, *F*/*F*_o_ was calculated with the following formula: (*F* − *F*_o_) / *F*_o_ and converted to *Z*-scores. Due to the nature of the behavior studied, a swivel is essential. We employed a rotatory-assisted photometry system that has no light path interruptions (Doric Lenses). In addition, black acrylic powder was mixed in the dental cement to assure that ambient light was not leaking into the implant and reaching the optical fiber; this was tested in each animal by comparing fluorescence signals in the dark versus normal cage illumination.

### Optogenetics

The implanted optical fibers were connected to patch cables using sleeves. A black aluminum cap covered the head implant and completely blocked any light exiting at the ferrule's junction. Furthermore, the experiments occurred in a brightly lit cage that made it difficult to detect any light escaping the implant. The other end of the patch cable was connected to a dual light swivel (Doric lenses) that was coupled to a green laser (520 nm; 100 mW) to activate Arch or a blue laser (450 nm; 80 mW) to activate ChR2. In experiments expressing Arch, green light was applied continuously at different powers (3.5, 7, 15, 25, and 35 mW). In experiments expressing ChR2, blue light was applied at the same power in different patterns, including continuous (Cont) and in trains of 1 ms pulses at different frequencies (2–100 Hz). The blue light included low (∼1 mW), medium (∼3 mW), and high (∼6 mW) powers. Unless otherwise noted, if the effects of low and medium blue light power were similar, the results were combined. Power is regularly measured by flashing the connecting patch cords onto a light sensor—with the sleeve on the ferrule.

During optogenetic experiments that involve avoidance procedures, we compared five different trial types: CS, CS + light, light, NoCS, and NoCS + light trials. “CS trials” were normal trials for a particular procedure without optogenetic light. CS* + *“light trials” were identical to CS trials except that optogenetic light was delivered simultaneously with the CS and US (if delivered) during the avoid and escape intervals, respectively. “Light trials” were identical to CS + light trials, but the CS was omitted to determine if the light could serve the same signaling function as the CS. “NoCS trials” were catch trials that lack CS or US to measure responses delivered by chance. “NoCS”* + *“light trials” are identical to NoCS trials, but light was delivered to determine its sole effects. To perform within-group RM comparisons, the different trial types for a procedure were delivered randomly within the same session. In addition, the trials were compared between different groups, including no-opsin mice that did not express opsins but were subjected to the same trials including light delivery.

### Video tracking

During experiments, mice are placed in standard shuttle box (16.1″ × 6.5″). All mice in the study were continuously video tracked (30–100 FPS) in synchrony with the procedures and other measures. We automatically tracked head movements with two color markers attached to the head connector—one located over the nose and the other between the ears. The coordinates from these markers form a line (head midline) that serves to derive several instantaneous movement measures per frame (Zhou et al., 2023). Overall head movement was separated into “rotational” and “translational” components (unless otherwise indicated, overall head movement is presented for simplicity and brevity, but the different components were analyzed). Rotational movement was the angle formed by the head midline between succeeding video frames multiplied by the radius. Translational movement resulted from the sum of linear (forward vs backward) and sideways movements. “Linear” movement was the distance moved by the ears marker between succeeding frames multiplied by the cosine of the angle formed by the line between these succeeding ear points and the head midline. “Sideways” movement was calculated as linear movement, but the sine was used instead of the cosine. Pixel measures were converted to metric units using calibration and expressed as speed (cm/sec). We used the time series to extract window measurements around events (e.g., CS presentations). Measurements were obtained from single-trial traces and/or from traces averaged over a session. In addition, we obtained the direction of the rotational movement with a “head angle” or “bias” measure, which was the accumulated change in angle of the head per frame (vs the previous frame) zeroed by the frame preceding the stimulus onset or event (this is equivalent to the rotational speed movement in degrees). The “time to peak” is when the “extrema” occurs versus event onset.

To detect spontaneous turns or movements from the head tracking, we applied a local maximum algorithm to the continuous head angle or speed measure, respectively. Every point is checked to determine if it is the maximum or minimum among the points in a range of 0.5 s before and after the point. A change in angle of this point >10° was a detected turn in the direction of the sign. We further sorted detected turns or movements based on the timing of previous detected events.

### Histology

Mice were deeply anesthetized with an overdose of isoflurane. Upon losing all responsiveness to a strong tail pinch, the animals were decapitated, and the brains were rapidly extracted and placed in fixative. The brains were sectioned (100 µm sections) in the coronal or sagittal planes. Some sections were stained using NeuroTrace or cresyl violet. All sections were mounted on slides, coverslipped with DAPI mounting media, and all the sections were imaged using a slide scanner (Leica Thunder). We used an app we developed with OriginLab (Brain Atlas Analyzer) to align the sections with the Allen Brain Atlas Common Coordinate Framework (CCF) v3 (Wang et al., 2020). This reveals the location of probes, fluorophores, and lesions versus the delimited atlas areas. We used it to delimit NeuroTrace or cresyl violet stained sections of electrolytic lesion mice and determine the extent of the lesions.

## Results

### NAc neurons code movement direction

**Figure 1.**
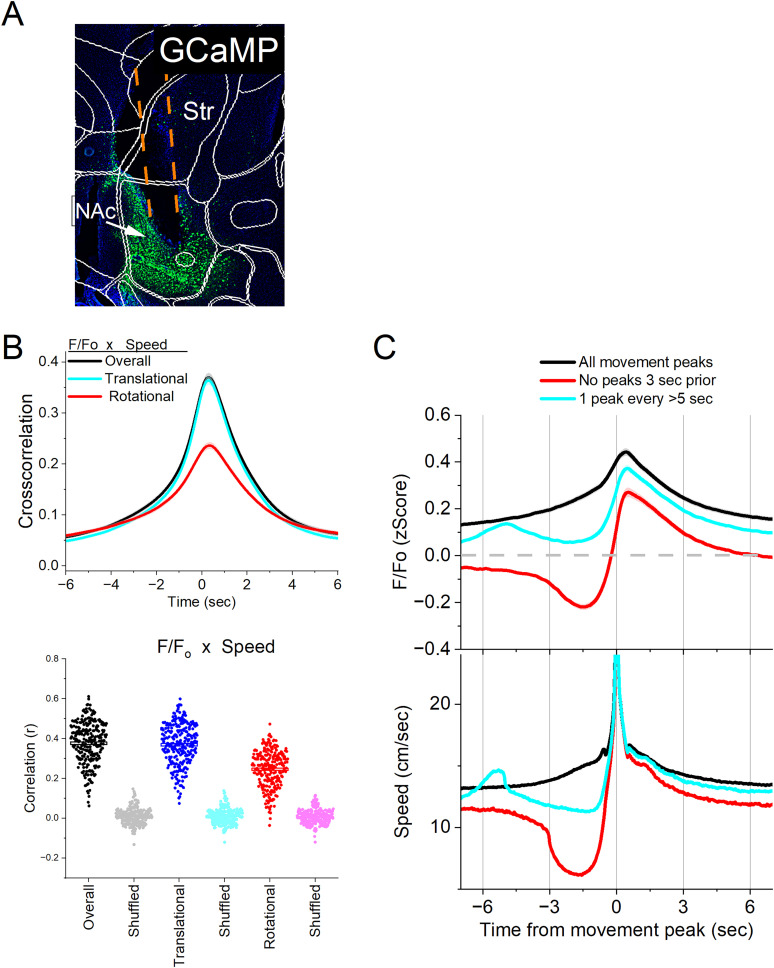
Calcium imaging fiber photometry reveals that NAc GABAergic neurons activate during spontaneous exploratory movement. ***A***, Parasagittal section showing the optical fiber tract reaching NAc and GCaMP7f fluorescence expressed in GABAergic neurons around the fiber ending. The sections were aligned with the Allen Brain Atlas. The right panel depicts the range of fiber endings for photometry and optogenetic experiments. OT, olfactory tubercle; AON, anterior olfactory nucleus; SI, substantia innominata; BST, bed nucleus of stria terminalis; Str, dorsal striatum. ***B***, Cross-correlation between movement and NAc *F*/*F*_o_ for the overall (black traces), rotational (red), and translational (blue) components top panel). Per session (dots) and mean ± SEM (rectangle) linear fit (correlation, *r*) between overall movement and NAc *F*/*F*_o_, including the rotational and translational components (bottom panel). The lighter dots show the linear fits after scrambling one of the variables (bottom panel, shuffled). ***C***, *F*/*F*_o_ calcium imaging time extracted around detected spontaneous movements. Time zero represents the peak of the movement. The top traces show *F*/*F*_o_ mean ± SEM of all movement peaks (black), those that had no detected peaks 3 s prior (red), and peaks taken at a fixed interval >5 s (cyan). The bottom traces show the corresponding movement speed for the selected peaks (*n* = 7 mice).

Since striatal medium spiny output neurons are GABAergic and activate concurrently during behavior, we targeted them as a coherent population using Vgat-Cre mice (Vong et al., 2011). To assess the population activity of NAc GABAergic neurons, we expressed GCaMP7f (Chen et al., 2013) in these neurons by locally injecting a Cre-AAV in Vgat-Cre mice (*n* = 7). After implanting a single optical fiber within the NAc, we employed calcium imaging fiber photometry, as previously described (Hormigo et al., 2021b; Zhou et al., 2023). Figure 1*A* illustrates a representative trajectory of the optical fiber, targeting GCaMP-expressing NAc GABAergic neurons. The estimated imaged volume extends ∼200 µm from the optical fiber ending, encompassing ∼2.5 × 10^7^ µm^3^ (Pisanello et al., 2019).

In *ad libitum* moving mice, we conducted continuous measurements of calcium fluorescence (*F*/*F*_o_) and spontaneous movement, while mice explored an arena. To establish the relationship between movement and NAc activation, we computed the cross-correlations between the continuous variables (Fig. 1*B*, top). Notably, overall movement was strongly correlated with NAc neuron activation. When the movement was dissociated into rotational and translational components, the cross-correlation predominantly involved the translational movement. A linear fit between the movement and *F*/*F*_o_ (integrating over a 200 ms window) revealed a strong linear positive correlation that was stronger for the translational component (Fig. 1*B*, bottom). These relationships were absent when one variable in the pair was shuffled (Fig. 1*B*, bottom).

To further evaluate the relation between movement and NAc activation, we detected spontaneous movements and time extracted the continuous variables around the detected movements peaks (Fig. 1*C*) following our previous studies in other regions (Hormigo et al., 2023; Zhou et al., 2023). The detected movements were classified in three categories. The first category includes all peaks (Fig. 1*C*, black traces), which revealed a strong NAc neuron activation in relation to movement. The second category includes movements that had no detected peaks 3 s prior (Fig. 1*C*, red traces), which essentially extracts movement onsets from immobility. There was a sharp activation in association with the movement onset. The third category sampled the peaks by averaging every >5 s to eliminate from the average the effect of closely occurring peaks (Fig. 1*C*, cyan traces). This category includes movement increases from ongoing baseline movement (not movement onsets from immobility) and showed a strong discharge of NAc neurons. For the three categories of movement peaks, the NAc activation around movement was significant compared with baseline activity (Tukey’s *p* < 0.0001). Thus, NAc neurons activate in relation to the occurrence of movement.

**Figure 2.**
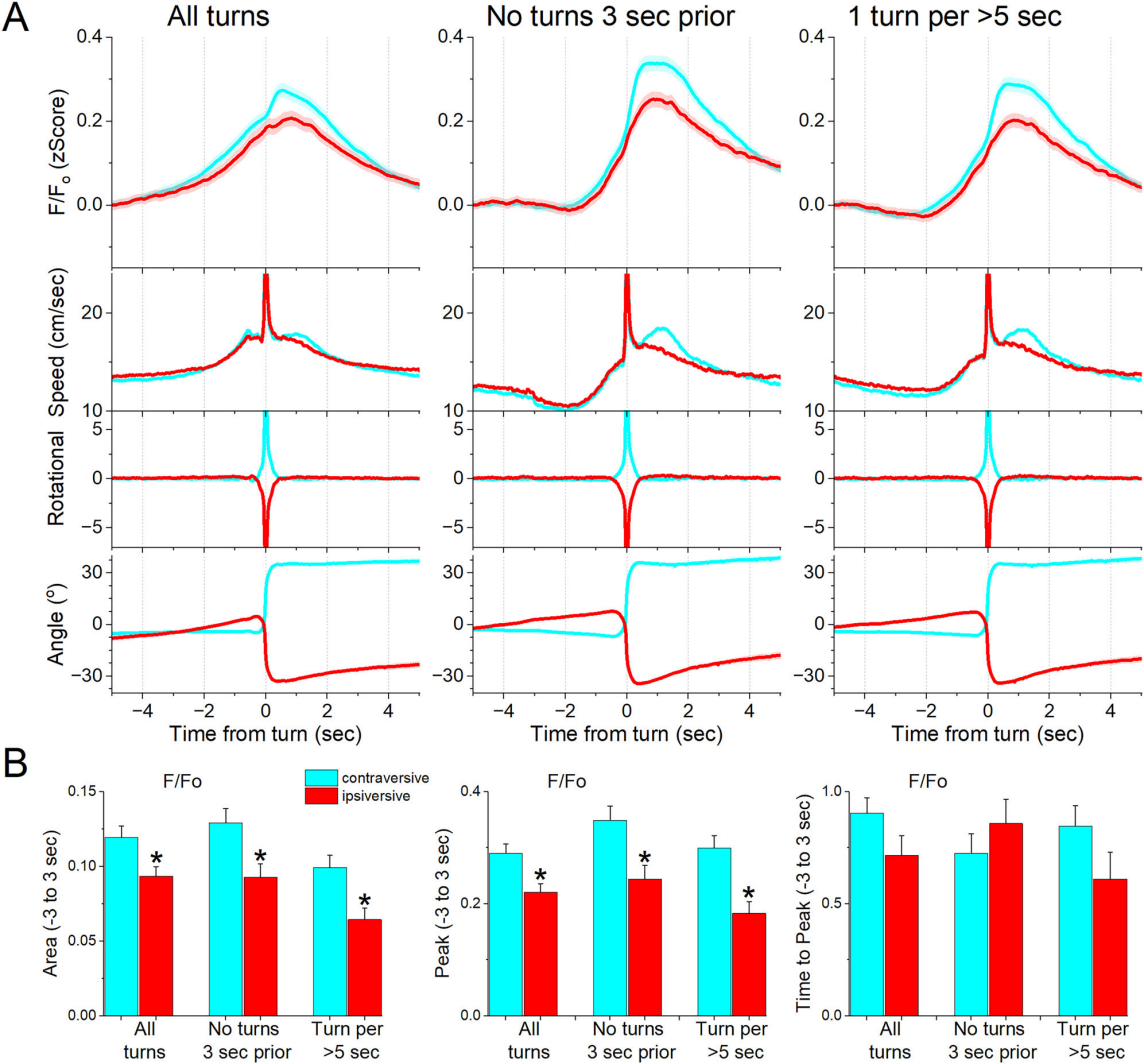
NAc GABAergic neurons code the direction of spontaneous contraversive exploratory turning movements. ***A***, *F*/*F*_o_ calcium imaging, overall movement, rotational movement, and angle of turning direction for detected movements classified by the turning direction (ipsiversive and contraversive; red and cyan) versus the side of the recording (implanted optical fiber). At time zero, the animals spontaneously turn their head in the indicated direction. The columns show all turns (left), those that included no turn peaks 3 s prior (middle), and peaks selected at a fixed interval >5 s (right). Note that the speed of the movements was similar in both directions. ***B***, Population measures (area of traces 3 s around the detected peaks) of *F*/*F*_o_ and movement (overall, rotational, and translational) for the different classified peaks. The measures that were different (*p* < 0.05) between ipsiversive and contraversive movements are indicated by * (*n* = 7 mice).

We then determined if the NAc activation depends on the ipsiversive or contraversive direction of the head movement. Figure 2*A* shows turns in the contraversive (Fig. 2*A*, cyan) and ipsiversive (Fig. 2*A*, red) directions in relation to the recorded NAc neurons. While the detected turns were opposite in direction and similar in amplitude, the NAc neuron activation was sharper when the head turned in the contraversive direction. We compared the area, peak amplitude, and peak timing of the *F*/*F*_o_ activation between ipsiversive and contraversive turns (Fig. 2*B*). For “all turns, no turns 3 s prior and one turn per >5 s”; the *F*/*F*_o_ area (Tukey’s *t*_(256) _= 6.16; *p* < 0.0001; Tukey’s *t*_(256) _= 4.99; *p* = 0.0005; and Tukey’s *t*_(256) _= 5.22; *p* = 0.0003) and amplitude (Tukey’s *t*_(256) _= 6.82; *p* < 0.0001; Tukey’s *t*_(256) _= 5.89; *p* < 0.0001; and Tukey’s *t*_(256) _= 6.4; *p* < 0.0001) of contraversive turns were larger than ipsiversive turns. However, the times to peak did not differ. Therefore, NAc GABAergic neurons code movement direction discharging more sharply to contraversive turns.

### NAc neurons activate during goal-directed avoidance movement

The results indicate that NAc neurons activate during movement. Therefore, we explored the activation of NAc neurons during a series of cued (signaled) avoidance procedures (AA1, AA2, AA3, and AA4; Zhou et al., 2022, 2023) in which mice move (actively avoid) or postpone movement (passively avoid) to prevent an aversive US. Since these procedures are signaled by tones, we first tested if auditory tones led to the discharge of NAc neurons during mapping sessions in *ad libitum* behaving mice.

**Figure 3.**
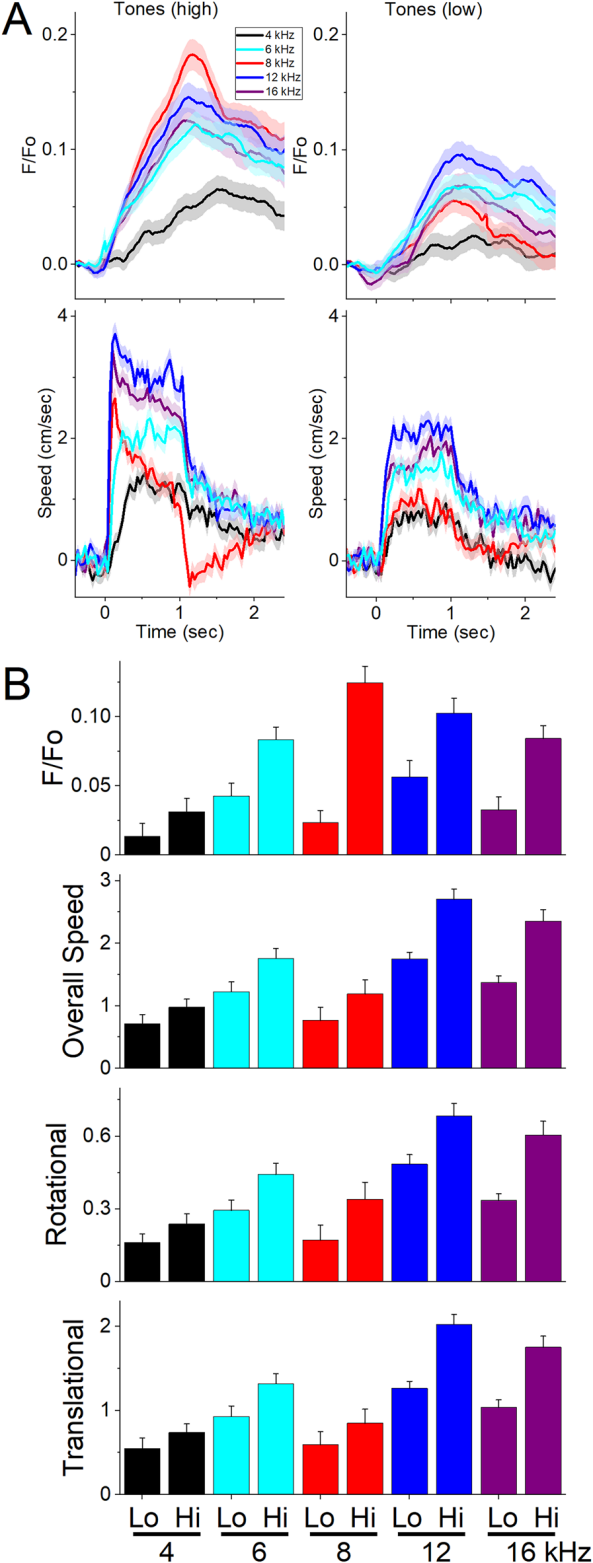
NAc GABAergic neurons discharge to auditory tones in association with movement. ***A***, Example *F*/*F*_o_ calcium imaging and movement traces (mean ± SEM) evoked from NAc neurons by auditory tones (1 s) of different saliency. The tones vary in frequency (4–16 kHz) and SPL (low and high dB). ***B***, Area of *F*/*F*_o_, overall movement, and movement components (rotational and translational) measured during a time window (0–2 s) after the tone onset (*n* = 7 mice).

Mice (42 sessions in seven mice; Fig. 3*A*,*B*) were placed in a small cage (half the size of a shuttle box), and 10 auditory tones of different frequencies (4, 6, 8, 12, 16 kHz) and saliencies, defined by SPL (in dB; low and high, ∼70 and ∼85 dB), were presented in random order (1 s tones every 4–5 s, each repeated 10 times per session). The NAc neuron discharge caused by the tones (0–1.5 s window) depended on SPL (2WayRMANOVA *F*_(1,41) _= 61.33; *p* < 0.0001) and frequency (*F*_(4,164) _= 10.05; *p* < 0.0001), and these factors interacted (*F*_(4,164) _= 5.53; *p* = 0.0003). In general, higher SPL and frequency tones produced stronger NAc activation (Fig. 3), but the lowest frequency (4 kHz) had a weaker activating effect that was not as sensitive to SPL as the higher frequencies (Tukey’s *t*_(164)_ = 1.9; *p* = 0.2 Lo vs Hi at 4 kHz). However, similar effects were observed on movement (overall speed). The movement evoked by the tones also depended on tone frequency (*F*_(4,164) _= 31.79; *p* < 0.0001) and SPL (*F*_(1,41) _= 76.81; *p* < 0.0001), and these factors interacted (*F*_(4,164) _= 3.94; *p* = 0.0044). Both the rotational and translational motion components (Fig. 3*B*) evoked by the tones showed effects like overall movement (SPL or frequency *p* < 0.0001). However, it is worth noting that the amplitudes of *F*/*F*_o_ activation (0.1–0.2 *Z*-score) and movements (∼2–4 cm/s) evoked by the tones are relatively small. The results show that salient auditory tones cause NAc neurons to discharge in association with movement.

**Figure 4.**
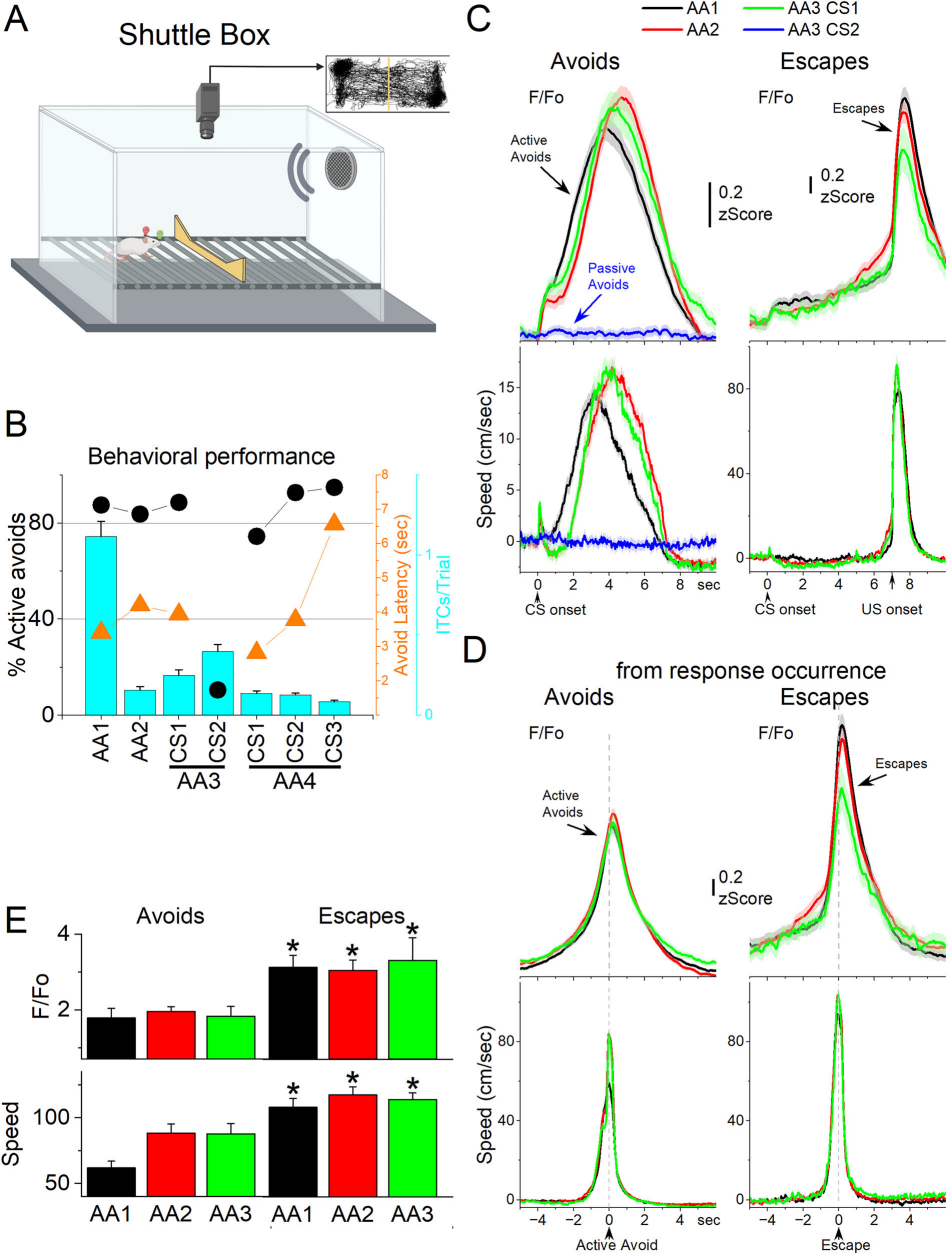
NAc GABAergic activation in the context of signaled active avoidance. ***A***, Arrangement of the shuttle box used during signaled avoidance tasks. ***B***, Behavioral performance during the four different avoidance procedures showing the percentage of active avoids (black circles), avoidance latency (orange triangles), and ITCs (cyan bars). ***C***, *F*/*F*_o_ and overall movement traces from the CS onset for AA1, AA2, and AA3 (CS1 and CS2) procedures per trials classified as avoids (left) or escapes (right) of CS-evoked orienting responses measured by tracking overall head speed. ***D***, Same as ***C*** from response occurrence. ***E***, Population measures of *F*/*F*_o_ and speed for avoids and escapes during AA1, AA2, and AA3 (CS1). Asterisks denote *p* < 0.05 versus avoids (*n* = 7 mice).

We then measured NAc neuron activation as the mice performed the avoidance procedures in a shuttle box (Fig. 4*A*). Figure 4*B* shows the behavioral performance of the animals in these tasks including the percentage of avoids (black circles), the avoid latencies (orange triangles), and the number of ITCs (cyan bars). As previously shown, animals perform a large percentage of active avoids during the AA1, AA2, AA3-CS1, and AA4 procedures (Hormigo et al., 2021b). During AA2, ITCs are punished. Consequently, avoid latencies shift longer in an apparent reflection of caution (Zhou et al., 2022). During AA3, animals learn to discriminate between two CS's, passively avoiding when AA3-CS2 is presented. During AA4, the avoid latencies adapt to the varying duration of three active avoid intervals (4, 7, and 15 s) signaled by different CS tones.

Figure 4*C* shows *F*/*F*_o_ and movement traces from the CS onset for the AA1 (black), AA2 (red), and AA3 (green) avoidance procedures classified as correct responses (left panel, avoids; for AA3-CS2 correct passive avoids are shown in blue) or errors (right panel, escapes). During the avoidance procedures, the CS that drives active avoids (Fig. 4*C*, black, red, green) caused a sharp and fast (<0.5 s) *F*/*F*_o_ peak at the CS onset, which is associated with the typical orienting head movement evoked by the CS depending on task contingencies and SPL (Zhou et al., 2023). AA3-CS2, which drives passive avoids, produced nil NAc activation at the CS2 onset in association with nil orienting head movement due to lower SPL. Thus, the CS onset NAc activation evoked by CS2 was smaller than the activation evoked by CS1 (Tukey’s *t*_(14)_ = 4.53; *p* = 0.0064).

The ensuing avoidance movement was associated with strong NAc neuron discharge (Fig. 4*C*, avoids). In AA1, there was a large *F*/*F*_o_ activation related to the avoidance movement. When mice transitioned to AA2, the NAc activation shifted right following the delayed avoid latencies typical of this procedure (Fig. 4*C*, black vs red traces). Thus, the activation of NAc neurons is closely associated with the active avoid movement. In addition, when mice failed to avoid, NAc neurons activated during the escape interval in relation to the fast escape responses evoked by the US (Fig. 4*C*, escapes). Furthermore, during the AA3 procedure, only CS1, which drives active avoids, produced strong NAc activation (Fig. 4*C*, green). CS2, which drives passive avoids, produced nil NAc activation (Fig. 4*C*, blue). To measure avoid and escape responses, we extracted the *F*/*F*_o_ and speed from the response occurrence for AA1, AA2, and AA3 (Fig. 4*D*). Both avoids and escapes were associated with robust NAc activation during the three procedures (Fig. 4*D*,*E*). However, since escapes occurred at higher speeds (Tukey’s *t*_(6)_ = 12.04; *p* = 0.0001 avoids vs escapes), they were associated with stronger NAc activation than avoids (Tukey’s *t*_(6)_ = 6.65; *p* = 0.0033). Moreover, as is typical (Zhou et al., 2022), the speed of avoids was higher during AA2 compared with AA1 (*t*_(12)_ = 4.85; *p* = 0.0129 AA1 vs AA2), but this was not consistently associated with a larger NAc activation during AA2.

**Figure 5.**
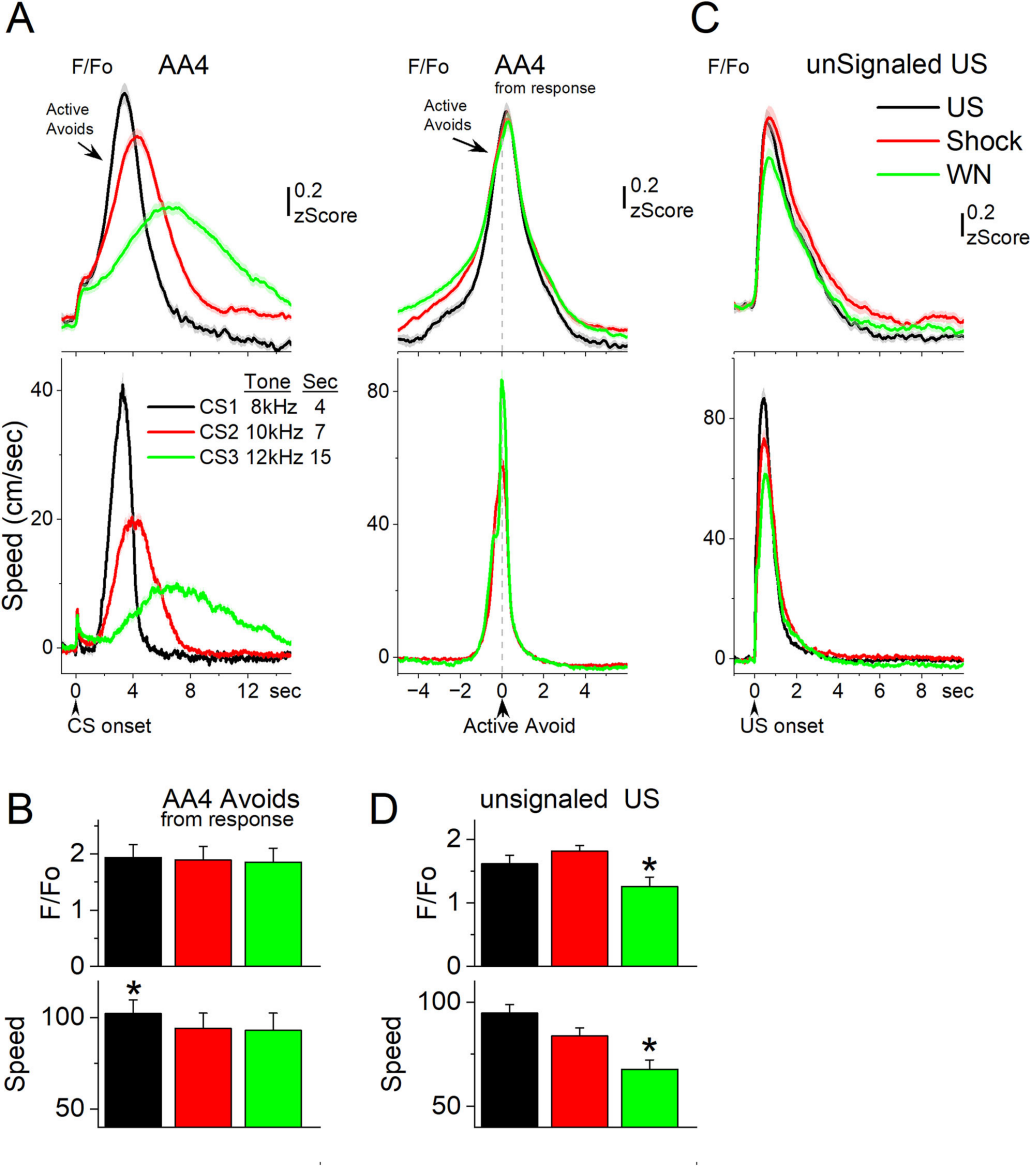
NAc GABAergic neurons track the avoidance and escape movement. ***A***, *F*/*F*_o_ and overall movement traces from the CS onset (left) and response occurrence (right) for avoids during the AA4 procedure, which include three CS’s that signal avoidance intervals of different durations. ***B***, Population measures (−3 to 3 s area, mean ± SEM) from response occurrence. Asterisks denote significant differences versus other stimuli. ***C***, *F*/*F*_o_ and overall movement traces from the US onset for escapes during the unsignaled US procedure, which includes the US, or each of its components delivered alone (footshock and white noise). ***D***, Population measures (0–5 s area, mean ± SEM) for the data in ***C***. Asterisks denote significant differences versus other CS's (*n* = 7 mice).

During AA4, mice adapt their avoidance movement to the duration of the avoidance interval signaled by each of the three CSs (Hormigo et al., 2021b). We found that NAc activation reflected the shift in the avoidance movement (Fig. 5*A*). When responses were measured from response occurrence (Fig. 5*A*, right panels, and 5*B*), the CS1 avoids speed was faster than CS2 (Tukey’s *t*_(36)_ = 5.3; *p* = 0.001) or CS3 (Tukey’s *t*_(36)_ = 5.2; *p* = 0.001), consistent with the more imminent threat signaled by CS1, which has a shorter 4 s avoidance interval. However, this was not reflected in a difference of the peak activation of NAc neurons (RMANOVA *F*_(2,36)_ = 0.72; *p* = 0.48).

Since mice have relatively few escapes (mostly avoids), we conducted additional sessions (Fig. 5*C*,*D*; seven mice) in which the US was presented unsignaled to drive an escape on every trial. To differentiate footshock and white noise contributions to the US activation, trials in the same session presented them alone. The unsignaled US evoked a strong NAc activation in association with the fast escapes. When the footshock was presented alone, the NAc activation and escapes resembled the full US (Fig. 5*C*,*D*). In contrast, the white noise alone drove escapes at lower speeds and less NAc activation than the footshock alone (Tukey’s *t*_(32)_ = 6.6; *p* = 0.00014 speed; Tukey’s *t*_(32)_ = 4.8; *p* = 0.004 *F*/*F*_o_) or the full US (Tukey’s *t*_(32)_ = 11.2; *p* < 0.0001 speed; Tukey’s *t*_(32)_ = 6.3; *p* = 0.0002 *F*/*F*_o_). Thus, the NAc discharge during US presentations is related to the speed of the escape movement.

In conclusion, NAc neurons activate at the CS onset in association with an orienting movement and then discharge more robustly during the ensuing active avoid and escape movements. NAc activation has the potential to drive active avoids, but NAc activation may instead reflect the ongoing movement.

### Direct inhibition of NAc neurons has little effect on avoidance behaviors

Since NAc GABAergic neurons activate robustly during signaled active avoidance and escape movements, we next determined the effect of directly optogenetically inhibiting NAc GABAergic neurons on signaled active avoidance.

**Figure 6.**
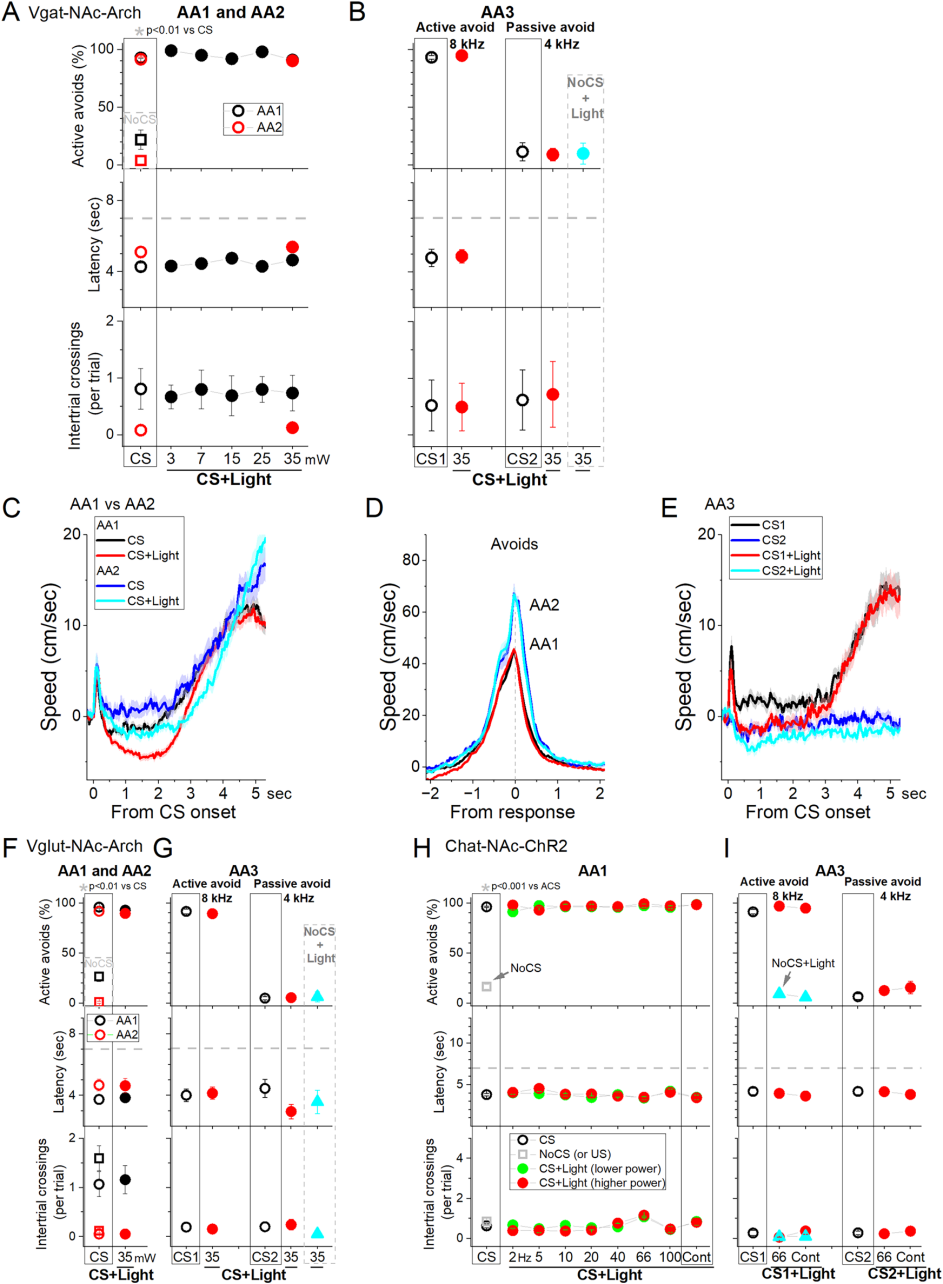
Optogenetic inhibition of NAc GABAergic neurons has little effect on signaled avoidance. ***A***, ***B***, Effect of Cont green light delivered at different powers on AA1/2 (***A***) and AA3 (***B***) in mice expressing eArch3.0 in NAc GABAergic neurons (*n* = 5 mice). ***C***, Traces of overall movement (speed) during AA1 or AA2 for CS trials, and CS + light trials combined. The trials are aligned by the CS onset, which reveals the orienting response evoked by the CS followed by the ensuing avoid action. Note that CS + light trials suppress ongoing movement prior to the onset of the avoid action without impairing the ability to respond. ***D***, Same as ***C*** but traces are aligned by the avoidance response occurrence, which reveals the peak speed of the response. Note that avoids are not different between CS and CS + light trials. As usual, AA2 produces faster avoids than AA1. ***E***, Same as ***C*** but for AA3. CS1 and CS2 are control trials for active avoidance and passive avoidance, respectively. As per AA1 and AA2, ongoing movement is suppressed by the light. ***F***, ***G***, Same as ***A*** and ***B*** but for a control group of mice that do not express Arch in GABAergic NAc neurons as they were injected with an AAV targeting glutamatergic neurons. The light has negligible effects on AA1, AA2 (***F***), and AA3 (***G***; (*n* = 4 mice). ***H***, ***I***, Effect of blue light on Chat-Cre mice that express ChR2 in NAc cholinergic neurons. Patterns of blue light were applied into NAc during AA1 (***H***) and AA3 (***I***). The light caused negligible effects on the performance of the mice. CS trials are control trials without light. CS + light trials include the CS and light patterns (trains of 1 ms pulses at the noted frequencies in Hz or Cont). During AA3, CS1 trials are active avoidance trials (like CS trials in AA1/2), and CS2 trials are passive avoidance trials. The plots show the percentage of active avoids, the response latency, and the ITCs per trial. NoCS trials are catch trials without CS or consequence (*n* = 5 mice).

To inhibit NAc GABAergic neurons, we expressed eArch3.0 in the NAc of Vgat-Cre mice with bilateral injections of a Cre-inducible AAV (Vgat-NAc-Arch, *n* = 5 mice; Fig. 6*A*). Cre is robustly expressed in the entire NAc of Vgat-Cre mice (Vong et al., 2011), ensuring that the eArch3.0 filled the rostrocaudal extent of the NAc. Optical fibers were implanted in the middle of the NAc to target both rostral and caudal regions of NAc with increasing green light powers to cover the entire NAc (Fig. 1*A*). In light trials, we tested the effects of five green light powers (AA1) or the higher light power (AA2, AA3). We found that, compared with CS trials, inhibiting NAc neurons in CS + light trials had little effect on the percentage of active avoids as a function of green light power during AA1, AA2, and AA3-CS1 (Fig. 6*A*,*B*). Active avoids were unaffected by the powers tested during AA1 (Fig. 6*A* black symbols; RMANOVA *F*_(5,30)_ = 2.17; *p* = 0.0841 CS vs CS + light), AA2 (Fig. 6*A*, red symbols; RMANOVA *F*_(1,8)_ = 1.03; *p* = 0.3397), or AA3-CS1 (Fig. 6*B*, left; Tukey’s *t*_(27)_ = 1.61; *p* = 0.66). Moreover, passive avoids were also unaffected during AA3-CS2 (Fig. 6*B*, right; Tukey’s *t*_(27)_ = 3.04; *p* = 0.16). There was no significant difference in avoidance performance (AA1–3) between the Vgat-NAc-Arch mice and no-opsin (*n* = 5) mice (2Way-mixed light × group; *F*_(1,8)_ < 2; *p* > 0.2 group or interaction for avoids, latency, or ITCs).

Movement tracking revealed that inhibition of NAc GABAergic neurons had little effect on the orienting response during AA1, AA2, or AA3-CS2 trials (Fig. 6*C–E*; Tukey’s *t*_(18)_ = 0.63; *p* = 0.66). However, movement was suppressed after the orienting response and prior to the occurrence of the avoidance response (Fig. 6*C*; Tukey’s *t*_(18)_ = 5.02; *p* = 0.0023 CS vs CS + light). Intriguingly, the ongoing movement pause caused by the light was not associated with impaired avoidance performance (Fig. 6*A*,*B*; avoids percentage and latency were unaffected). Thus, during the CS + light trials, mice pause right after the orienting response but avoid rapidly. There is no significant change in avoid peak speed measured from avoid occurrence (Fig. 6*D*; Tukey’s *t*_(18)_ = 1.92; *p* = 0.47 CS vs CS + light from avoid occurrence).

As per the ChR2 experiments, we also tested control mice in which we injected the same Arch AAV in Vglut2 mice (*n* = 2) or an Arch AAV with a CaMKII promoter in C57BL/6J mice (*n* = 2) and combined these mice into a single control group (Fig. 6*F*,*G*; Glut-NAc-Arch; *n* = 4 mice). In these mice, we found no effect of green light on active avoids during AA1 (Fig. 6*F*, black symbols; RMANOVA *F*_(5,25)_ = 1.63; *p* = 0.18), AA2 (Fig. 6*F*, red symbols; RMANOVA *F*_(1,6)_ = 1.56; *p* = 0.25), and AA3-CS1 (Fig. 6*G*, left; Tukey’s *t*_(28)_ = 2.55; *p* = 0.39). We also found no effect on passive avoids during AA3-CS2 (Fig. 6*G*, right; Tukey’s *t*_(28)_ = 0.21; *p* = 0.99) or NoCS + light trials.

Since medium spiny GABAergic output neurons in NAc are controlled by cholinergic interneurons, which can inhibit NAc output when activated (de Rover et al., 2002; Castro and Bruchas, 2019), we expressed ChR2 by injecting an AAV in the NAc of Chat-Cre mice (Chat-NAc-ChR2; *n* = 5). The results revealed little effect of exciting cholinergic NAc neurons with blue light on active avoids during AA1 (Fig. 6*H*, RMANOVA *F*_(16,48)_ = 1.28; *p* = 0.2462), AA2 (RMANOVA *F*_(8,24)_ = 2.29; *p* = 0.05), and AA3-CS1 (Fig. 6*I*; Tukey’s *t*_(9)_ = 1.31; *p* = 0.79) or on passive avoids during AA3-CS2 (Fig. 6*I*; Tukey’s *t*_(9)_ = 2.49; *p* = 0.34). Since these neurons are not known to project outside of NAc, the results indicate that the suppression of active avoidance caused in Vgat-NAc-ChR2 mice may be due to inhibitory effects outside of NAc.

Taken together, these results indicate that inhibiting NAc neurons does not impair signaled active or passive avoidance behaviors.

### NAc lesions have little effect on avoidance behavior

**Figure 7.**
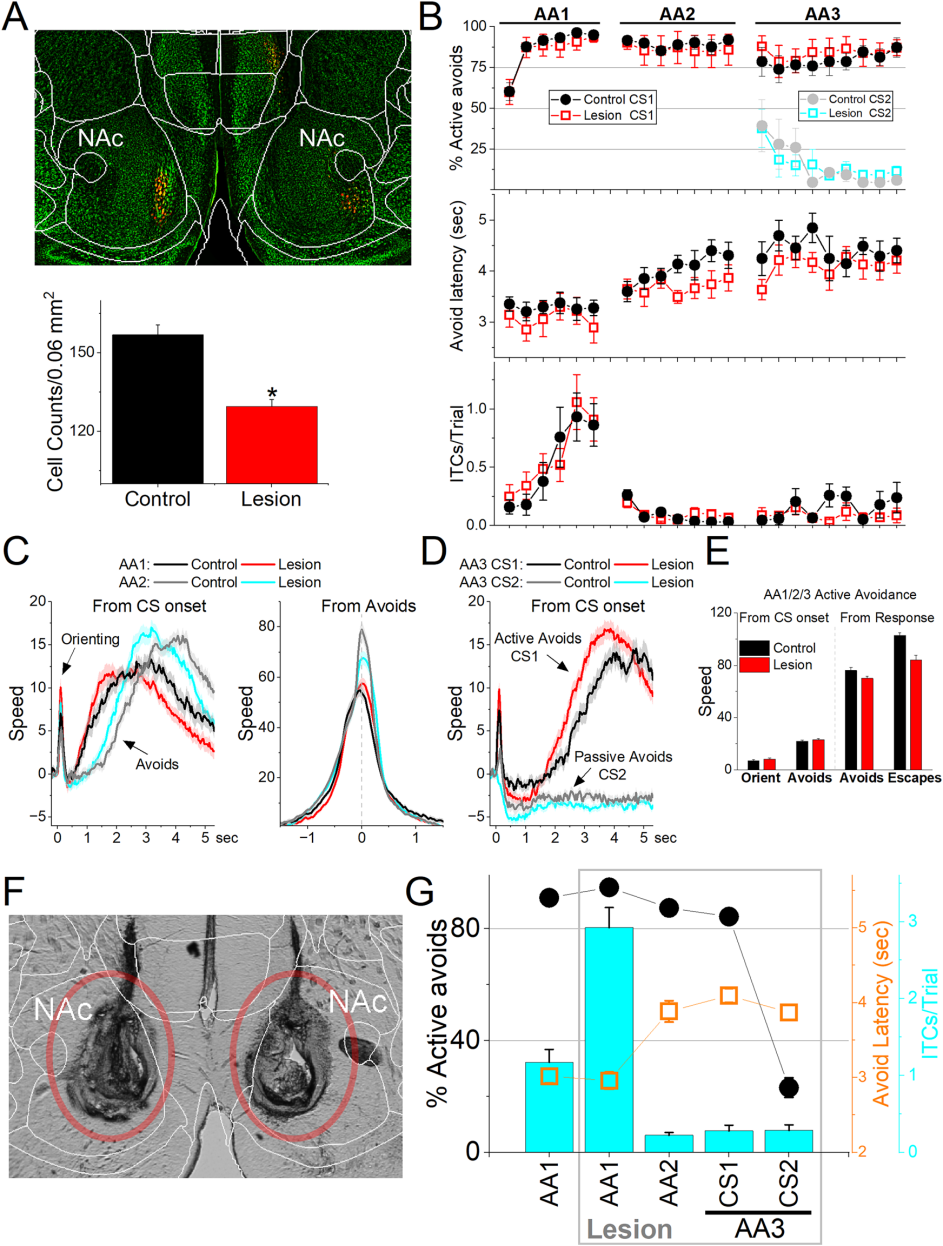
Lesions of NAc GABAergic neurons do not impair signaled avoidance learning or performance. ***A***, Coronal NeuroTrace (green) stained section of a Vgat-Cre mouse injected with a Cre-dependent AAV-dtA in the NAc to kill GABAergic neurons. We counted the number of cells in the NAc in controls and lesion mice. There was a significant reduction in the number of NAc neurons in the lesion mice. ***B***, Behavioral performance during learning of AA1, followed by AA2 and AA3 procedures showing the percentage of active avoids (top), avoid latency (middle), and ITCs (bottom) for control and lesion mice. The AA3 procedure shows CS1 and CS2 trials for the same sessions. Active avoids during AA3-CS2 trials are errors, as the mice must passively avoid during CS2. Lesion mice tended to have shorter avoid latencies, but as is usual both control and lesion mice shifted their avoid latencies longer during AA2 compared with AA1 (*n* = 9 lesion mice; *n* = 6 control mice). ***C***, Movement (speed) from the CS onset (left) and from avoid occurrence (right) during AA1 and AA2 procedures for control and lesion mice. The lesion had little effect on movement other than the tendency to shift avoid movement sooner. However, the overall avoid speed measured from response occurrence was not faster in lesion mice. As typically observed in controls, lesion mice avoided faster during AA2 compared with AA1. ***D***, Same as ***C*** for AA3. ***E***, Population measures of orienting, avoidance, and escape responses from the CS onset (left) and from response occurrence (right) for overall movement. ***F***, Bilateral electrolytic lesions targeting the NAc. ***G***, Effect of bilateral electrolytic NAc lesions on behavioral performance in a RM design. The plot shows the percentage of active avoids (filled black circles), avoid latency (open orange squares), and ITCs (cyan bars). Mice were trained in AA1 prior to the lesion and then placed back in AA1, followed by AA2 and AA3. The main effect of the lesion was to increase the number of ITCs during AA1, with little negative effect on active avoids. During AA2, lesion mice normally abolished their ITCs, which are punished, and shifted avoid latencies longer with little effect on avoidance performance. During AA3, lesion mice also performed AA3 normally, actively avoiding during CS1 and passively avoiding during CS2 (*n* = 9 lesion mice).

To determine if NAc neurons are important for learning and/or performing avoidance behaviors, we bilaterally injected AAV8-EF1a-mCherry-flex-dtA into the NAc of Vgat-cre mice (*n* = 9) to lesion NAc GABAergic neurons. We counted the number of neurons (NeuroTrace) in the NAc lesion and control mice to verify the lesion and found that the AAV injection reduced the number of NAc neurons in sections from the full extent of NAc, including its rostral and caudal portions (Fig. 7*A*; Mann–Whitney *Z* = 4.99; *p* < 0.0001 lesion vs control); this included the shell region core regions, both of which showed significant reductions in cell counts. However, this had little effect on the ability of mice to learn and perform signaled active avoidance tasks (Fig. 7*B*). Mice learned AA1 like control mice. There was no difference in the percentage of active avoids between control and lesion mice during the AA1 (ANOVA *F*_(1,13)_ = 0.03; *p* = 0.85 lesion vs control), AA2 (*F*_(1,13)_ = 0.02; *p* = 0.89), or AA3-CS1 (*F*_(1,13)_ = 0.53; *p* = 0.48). There was also no difference in either avoidance latency (*F*_(1,13)_ = 1.58; *p* = 0.23) or ITCs (*F*_(1,13)_ = 0.03; *p* = 0.85) between control and lesion mice during these active avoidance trials. Moreover, there was no difference in the percentage of passive avoids in AA3-CS2 (*F*_(1,13)_ = 0; *p* = 0.96) between control and lesion mice.

We also compared movement (overall, rotational, and translational speed) during task performance and found no significant differences between lesion and control mice. We combined the results for the three procedures (AA1, AA2, and AA3 tasks) because the effects were similar (Fig. 7*C*–*E*). The orienting response evoked by the CS1 onset was not different between control and lesion mice (Fig. 7*C*,*E*; ANOVA *F*_(1,13)_ = 0.06; *p* = 0.80, including the rotational component *F*_(1,13)_ = 0.12; *p* = 0.73). The active avoidance movement speed was not different between control and lesion mice (Fig. 7*C*,*E*; *F*_(1,13)_ = 0.4; *p* = 0.53). This applied to both the translational (*F*_(1,13)_ = 0.48; *p* = 0.49) and rotational (*F*_(1,13)_ = 0.01; *p* = 0.90) movement. Similarly, when avoids failed, the escape response movement was not different between control and lesion mice (*F*_(1,13)_ = 0.39; *p* = 0.54). Finally, the movement during passive avoids in AA3-CS2 trials did not differ between control and lesion mice (Fig. 7*D*; *F*_(1,13)_ = 0.33; *p* = 0.57). In conclusion, selective lesions of NAc GABAergic neurons had little effect on CS-evoked goal–directed movement.

Since the AAV-based lesion may leave some NAc cells intact, we performed electrolytic lesions of NAc (Fig. 7*F*), which assures elimination of NAc cells. The lesioning electrode was inserted into both rostral and caudal portions of NAc resulting in damage to the full rostrocaudal extent of the NAc. We tested the effect of the lesion in trained mice (*n* = 9; Fig. 7*G*) using an RM design. The lesion did not affect the percentage of active avoids (*F*_(1,8)_ = 0.61; *p* = 0.45) or avoid latency (*F*_(1,8)_ = 0.31; *p* = 0.59) in AA1. However, there was a large increase in the number of ITCs (*F*_(1,8)_ = 22.9; *p* = 0.001), which can lead to spurious avoids. Subsequent training of mice in AA2, which abolishes ITCs and eliminates spurious avoids, revealed that mice continued to perform avoids at high rates (∼88%) without ITCs. Furthermore, the lesion mice learned AA3 like normal animals, which require discriminating between CS1 and CS2 and selecting the appropriate action (Fig. 7*G*). These results indicate that NAc is not required to learn or perform active avoidance or to postpone action during passive avoidance. Brain processes such as decision-making (Go/NoGo in AA1–3), response inhibition (unsignaled and signaled passive avoidance in AA2/3), and stimulus discrimination (AA3) in the context of our procedures are not strongly impacted by NAc lesions.

## Discussion

We found that NAc neurons encode distinct facets of goal-directed movement during signaled avoidance. Calcium imaging showed that NAc GABAergic neurons activate during rotational exploratory movement, discerning the movement's direction by exhibiting heightened discharges during contraversive head turning. Additionally, these neurons robustly discharge during signaled active avoidance behaviors, predominantly characterized by translational movement. Thus, NAc neurons discharge during CS-evoked orienting and active avoidance movement suggesting a pivotal involvement of NAc in signaled avoidance actions. Consequently, we employed optogenetics and lesions to inhibit the activity of NAc GABAergic neurons.

To determine the necessity of NAc during signaled avoidance, we directly inhibited NAc GABAergic neurons using Arch. Interestingly, this manipulation resulted in minimal impact on signaled avoidance despite a notable decrease in ongoing movement. Furthermore, electrolytic or AAV-based lesions of NAc demonstrated little effect on signaled avoidance. In summary, our findings indicate that while NAc neurons represent various facets of behavior related to movement, they do not play a central role in the generation of signaled avoidance.

### NAc neurons represent orienting and goal-directed avoidance movements

Taken together, the results indicate that NAc neurons are activated by movement, aligning with prior studies indicating that both direct and indirect striatal neurons typically activate during movement but are silent in the absence of movement (Cui et al., 2013; Tecuapetla et al., 2014; Barbera et al., 2016). Striatal neurons continuously code movement variables, such as speed (Markowitz et al., 2018; Sales-Carbonell et al., 2018). This may be driven by their sensitivity to somatosensory input (Carelli and West, 1991; Robbe, 2018).

NAc GABAergic neurons exhibited activation linked to exploratory movement, discerning orientation direction by discharging more strongly during contraversive turns. Notably, during auditory mapping sessions, these neurons discharged in response to auditory stimuli devoid of predictive behavioral contingencies. However, stimuli activating NAc neurons consistently coincided with movement events. This includes the orienting reflex, characterized by a rapid (<300 ms) head movement in response to salient auditory stimuli (Sokolov, 1963; Zhou et al., 2023). Beyond the swift onset activation of NAc neurons triggered by auditory tones, additional activation persisted throughout the 2 s duration, associated with pronounced exploratory rotational movements induced by the tones, particularly contraversive motions.

In the context of signaled avoidance, calcium imaging showed that NAc GABAergic neurons activate at the CS onset in association with the orienting response but then discharge more robustly during the ensuing active avoidance and escape movements. NAc activation has the potential to drive active avoids, but NAc activation may instead reflect the ongoing movement, similar to other brain regions like the zona incerta (Hormigo et al., 2023). These findings highlight the important distinction between encoding and generating behavior: while broad brain regions may encode particular behaviors, only specific interconnected regions are likely essential for actually generating those behaviors.

Our behavioral procedures tested the relation between goal-directed behavior and NAc neuron activity. In general, during active avoidance movements, NAc neurons discharged strongly. In contrast, during passive avoids driven by auditory stimuli, which are not associated with movement, NAc neurons did not activate. Moreover, fast escape responses caused by unsignaled US presentations showed strong NAc activation, with the footshock evoking most of the activation, as the auditory white noise contributed little. Thus, regardless of the sensory modality driving the movement, NAc neurons activate in response to movement.

An interesting question is whether direct and indirect pathway NAc neurons activate distinctly during avoidance. Further imaging studies that target these two distinct NAc populations separately would be required to answer this question directly. One possibility is that direct pathway NAc neurons, traditionally associated with movement, would activate during active avoidance, while indirect pathway NAc neurons, traditionally associated with movement inhibition, would activate during passive avoidance. If this were the case, we would expect to see activation in NAc during passive avoidance reflecting the indirect pathway neurons. However, the only activation we observed was related to movement during active avoidance or other contexts, indicating that these cell groups may activate coherently during avoidance movement.

### NAc neurons are not required for goal-directed signaled avoidance

Direct inhibition of NAc GABAergic neurons with Arch in Vgat-NAc-Arch mice had little effects on signaled avoidance, although it significantly suppressed ongoing movement. Moreover, activation of ChR2-expressing GABAergic Chat interneurons, which do not project outside of the NAc (Castro and Bruchas, 2019), had minimal impact on signaled avoidance like direct inhibition with Arch. Lesions affecting the striatum, including the NAc, can either enhance or impair active avoidance behaviors (Kirkby and Kimble, 1968; Darvas et al., 2011; Bravo-Rivera et al., 2014; Lichtenberg et al., 2014; Wendler et al., 2014; Ramirez et al., 2015). The striatum's involvement in active avoidance has been recognized in both human and rodent studies (Oleson et al., 2012; Bravo-Rivera et al., 2015; Boeke et al., 2017). Our study agrees that NAc is engaged by active avoidance by coding aspects of the avoidance movement, including contraversive orienting. However, our study reveals that neither signaled active nor passive avoidance responses were affected by the direct inhibition or lesions of NAc GABAergic neurons. Although direct inhibition led to a marked suppression of ongoing movement, it did not impair the mice's ability to execute active avoidance responses. Our testing employed various goal-directed avoidance tasks that engage distinct neural processes, including decision-making (Go/NoGo in AA1–3), action inhibition (unsignaled and signaled passive avoidance in AA2–3), and stimulus discrimination (AA3). The lesions or optogenetic inhibition demonstrated minimal impact on the mice's proficiency in successfully performing these tasks.

Collectively, these findings suggest that NAc neurons do not play a critical role in either signaled active or passive avoidance behaviors. This aligns with the observation that the output of the basal ganglia through SNr is not inherently essential for mediating these behaviors under normal circumstances (Hormigo et al., 2021c). By design, our study targeted the entire NAc for inhibition or lesioning without separating among direct and indirect pathway neurons or sectors. It is possible that inhibiting or lesioning only one output pathway or NAc sector may produce deficits in avoidance behavior. In this case, it would be important to verify if this is caused by dysregulated, abnormal activity arising from the remaining pathway or sector. Thus, an important consideration is that while NAc is not requisite for normal avoidance behaviors, its dysregulation leading to aberrant output may significantly disrupt these behaviors by inhibiting its various targets, particularly in the midbrain. This may be relevant in the context of brain disorders, such as anxiety disorders leading to abnormal avoidance responses, and movement disorders disrupting signaled motions.

## Synthesis

Reviewing Editor: Arvind Kumar, KTH Royal Institute of Technology Decisions are customarily a result of the Reviewing Editor and the peer reviewers coming together and discussing their recommendations until a consensus is reached. When revisions are invited, a fact-based synthesis statement explaining their decision and outlining what is needed to prepare a revision will be listed below. The following reviewer(s) agreed to reveal their identity: Daniel Castro, T Chase Francis. Note: If this manuscript was transferred from JNeurosci and a decision was made to accept the manuscript without peer review, a brief statement to this effect will instead be what is listed below.

Synthesis

In this manuscript authors have analysed how neurons in the nucleus accumbens may contribute to avoidance and escape behaviors. The behavioral analysis is very carefully done and authors have tested their results using both optogenetic and lesion methods.

While both reviewers have appreciated that the authors have addressed an interesting question but both noted that the way the experiments have been designed we can only learn about the role of GABAergic neurons and there is no way in the current state to determine the role of different types of GABAergic and/or projection neurons - something essential for interpreting the role of NA in avoidance and escape behavior. However, we also understand that now the new experiments to isolate the role of different neuron types (especially projection neurons) is perhaps not feasible. Therefore, reviewers insist that the conclusions of the manuscript are revised accordingly to account for the limitations of the experimental design.

The detailed comments from the reviewers are appended below. We look forward to reading your revision.

Reviewer #1

In this manuscript by Zhou et al., the authors seek to understand how nucleus accumbens contributes to avoidance behaviors. To achieve this, they use a combination of fiber photometry, lesion, and optogenetic approaches. The find that while nucleus accumbens can modulate orienting behaviors, it is not actually necessary for generating avoidance behaviors. The authors use an elegant behavioral paradigm to distinguish between avoidance and escape behaviors.

Major Considerations:

1. Considering that the authors acknowledge that the NAc MSNs can be functionally and anatomically dissociated, it seems like a missed opportunity to not have done a direct versus indirect pathway genetic approach.

2. The authors analyzed the photometry data very thoughtfully and rigorously.

3. The lack of effect from the opto and lesion experiments is very interesting, and points to an important difference between encoding and generation.

4. The authors reference some ChR2 results, but there does not appear to be any text related to those experiments. I assume this is related to a prior version of the manuscript. If cleaned up, then the flow and impact of the manuscript would be significantly improved.

Reviewer #2

Zhou et al. show GABAergic neurons within the Nucleus Accumbens show increased calcium activity in response to movement, active avoidance, and escapes and are sensitive to cued motivated response. The authors show data that excitation, inhibition, or lesions of all GABAergic cells has little effect on avoidance behavior. While there is little concern about the experimental procedures, the use of Vgat-cre mice obscures interpretation of how output neurons may facilitate active avoidance outcomes and diminishes the ability to compare this research to other literature. However, the studies stand on their own with some alteration in interpretation and discussion. See individual comments below:

1. Since the NAc core and shell produce different behavioral outcomes, please specify the location of the fiber tracts for the majority of the animals. The representative image in Fig. 1 should contain labels for regions to make the location clearer.

2. It is unclear how the F/Fo and Speed graphs for all figures were averaged. The error bars are very small indicating a large number of movement events were captured. Please specify how averaging was conducted. In figure 3, were only movement related events included in the analysis?

3. Group N must be included for all analyzed figures and individual points need to be provided for each bar graph. It is unclear whether 7 mice were used in each experiment or if the same mice were used across all experiments.

4. Light powers of 35mW can cause artifactual changes to neurons caused by tissue heating or damage (PMID: 26166563). To claim that cells were inhibited by this light power a between groups control (eYFP) is necessary to substantiate this claim.

5. One of the primary concerns that should be discussed in the conclusions is the extensive number of GABAergic cell subtypes in the NAc. Vgat would encompass medium spiny neurons as well as fast spiking interneurons, low threshold spiking neurons, and other subtypes. Despite the low percentage of these cells, these interneurons have large effects on circuit behavior. Because of this, discussion about direct or indirect pathways and output selection to midbrain areas may not have relevance in the discussion.

Minor

1. Figure 4D is missing x-axis labels

## Author Response

Response to reviewer 2 and synthesis Synthesis: As we can see in Figure 7A, there is only a 20% reduction in the cell count. Based on this it is notjustified to claim that 'entire NAc was' targeted. Therefore we would like you to adjust your conclusions and not use phrases like "our study targeted the entire NAc". Reviewer 2: If the authors want to continue to claim that the entire NAc is not involved in this behavior, then a lesion of the entire NAc must be performed. Our study employs four distinct and complementary methods to rigorously conclude that the NAc is not required for this behavior. Few studies, to our knowledge, have utilized such a comprehensive range of techniques to support their conclusions. It is accurate to state that "our study targeted the entire NAc," as we specifically aimed to affect the full extent of the NAc, including both core and shell regions, using the following approaches: 1) Direct optogenetic inhibition using Arch. 2) Indirect optogenetic inhibition via excitation of ChR2-expressing GABA interneurons. 3.) Lesions with AAV-dtA. 4) Electrolytic lesioning. These methods were consistently applied across the full extent of the NAc. Regarding the cell count reduction in Figure 7A, the noted reduction likely underestimates actual cell death, as we did not employ specific markers for cell death or degeneration. Notably, this same AAV-dtA lesion approach, when applied to another brain region critical for avoidance behavior, produces a similar reduction in cell counts but effectively abolishes avoidance behavior. Additionally, cell counts in both core and shell regions of the NAc showed significant reductions that did not differ between each other, reinforcing the extent of the targeting. This is now indicated in the text. Importantly, electrolytic lesions-a technique that physically burns the affected areas- confirmed that even when the lesions undoubtedly spanned the entire NAc, there was little effect on avoidance behavior. This result is also significant because much smaller electrolytic lesions in behaviorally relevant brain regions strongly suppress avoidance behavior. Thus, the conclusions drawn from our study rest on a solid and consistent interpretation of the evidence from multiple experimental approaches. We believe our data strongly supports the assertion that the NAc, as a whole, is not essential for this specific behavior.
